# Purine Scaffold in Agents for Cancer Treatment

**DOI:** 10.1021/acsomega.5c00340

**Published:** 2025-04-29

**Authors:** Zdeněk Wimmer

**Affiliations:** † University of Chemistry and Technology in Prague, Department of Chemistry of Natural Compounds, Technická 5, CZ-16610 Prague 6, Czech Republic; ‡ Institute of Experimental Botany of the Czech Academy of Sciences, Isotope Laboratory, Vídeňská 1083, CZ-14220 Prague 4, Czech Republic

## Abstract

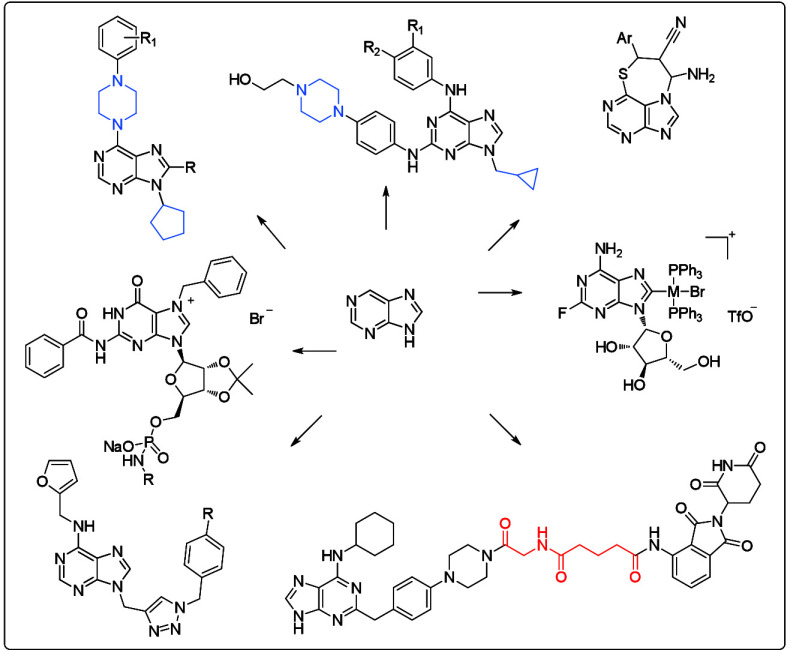

Cancer represents one of the most important and often fatal threats in the human population. Regarding the natural products, the purine scaffold appears in the purine bases in nucleic acids. Purine and its natural derivatives display a number of pharmacological effects. Previous investigations revealed that different compounds bearing the purine scaffold in their molecules belong to a group of potent agents for cancer treatment. Therefore, this review focuses on summarizing recently designed agents for potential cancer treatment bearing the purine scaffold as the key structural motif in the molecules. The reviewed structures clearly show the advantages and disadvantages of different substituents of the key scaffold that affect the final cytotoxic effects of the studied structures. The structure–activity relationship analysis shows a summary of different but potent compounds mentioned in this review and identifies the compounds receiving priority importance due to their high cytotoxicity and exceptional physicochemical characteristics. The effects of metal coordination, the formation of convenient conjugated molecules, and supramolecular self-assembly resulting in the production of biologically active nanovesicles and other nanoassemblies are also demonstrated. The reviewed original studies clearly showed the possible advantages of (a) metal ion coordination, (b) the formation of conjugates, and (c) designing smart and biocompatible nanoassemblies for biological activity in comparison with the characteristics of the parent compounds. This review is based on the most recent articles published in the last two years, 2023–2024, and it represents work with a highly interdisciplinary nature. Even if these original articles are not too numerous within the given period, the investigations published therein have clearly documented the importance of the purine scaffold in pharmacology and in medicinal and supramolecular chemistry.

## 1 Introduction

Even though purine itself was discovered and synthesized already in the 1860s, purine bases were isolated from nucleic acids much later.^1,2^ Besides the purine bases adenine and guanine, other nucleic acid bases are pyrimidine ones, i.e., cytosine, thymine, and uracil. The purine and pyrimidine bases participate in the formation of hydrogen bonds in the nucleic acid helixes. Purine and its analogues are capable of acting as possible inhibitors of enzymes, namely, phosphodiesterases, protein kinase (p38a), cyclin-dependent kinases (CDKs), sulfotransferases, HSP90 protein, MAP kinase, nonreceptor tyrosine kinase SRC, or protein kinase Clk. They display effects as antimicrobial, antifungal, antiviral, antihyperglycemic, and cytotoxic agents, and they act as immunostimulators, which were reviewed recently.^3,4^ Nevertheless, novel purine derivatives have been synthesized and studied since the most recent reviews were published in 2023 and 2024.^4−7^ All these reviews gave enough basic items of information on purine and its natural derivatives and summarize important bioactive molecules bearing the purine scaffold as the key structural motif.^4−7^ The reviews also show structures bearing the purine scaffold as ligands for coordination of metal ions and/or conjugation with other types of molecules capable of nanoassembling.^4−7^

One of the most recent papers summarized the action of purine-based compounds on various cancer targets;^6^ another one, published by the same team of authors, showed the major attributes of cancer affected through different cellular pathways and a list of different diseases that might be treated with purine-based therapeutic agents.^7^ Useful figures suggesting the location and importance of different structural motifs on the biological activity demonstrate the advantages of combining the purine scaffold with other structural motifs in designing novel agents with a potential anticancer effect.^7^

The objectives of this review are closely connected with the general intensity of investigation of the purine scaffold that has demonstrated its importance in the search for novel agents showing a cytotoxic effect. The present review also focuses on purine conjugation with other molecules. The reviewed structures were capable of self-assembling and coordinating metal ions. The present review also focuses on the structure–activity relationship analysis that has been based on the data published in the original papers and on the evaluation of the effects of conjugation with other molecules, often combined with the ability to coordinate metal ions. The structure–activity relationship analysis demonstrated enhancement of the cytotoxic effect of the target derivatives bearing the purine scaffold. This review has covered the field of investigation that has been of a highly interdisciplinary nature. The text is divided into several sections based on the structural motifs applied in the modification of the purine scaffold, even if this sorting of the reviewed compounds was neither easy nor fully satisfactory due to the fact that multiple structural motifs used by different authors often overlapped.

## 2 Purine Derivatives Bearing the Motif of Piperazine Partly Combined with Other Alicyclic Motifs

**Figure 1:**
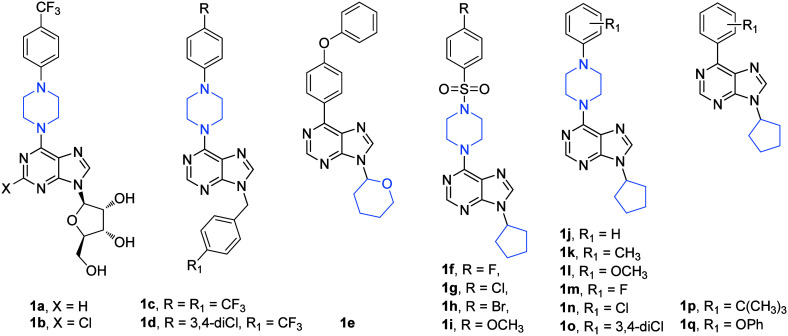
Structures of compounds **1a**–**1q**.

The cytotoxic effect of novel purine derivatives has been intensively studied by Turkish authors. They focused their attention on purine-based structures decorated by the piperazine motif.^8−10^ This structural motif has often appeared in biologically active structures studied previously.^11,12^ The authors used it in their series of purine derivatives represented by the compounds **1a**–**1d** (Figure 1) at the very beginning.^11,12^ The piperazine motif was substituted by the tetrahydropyrane motif in compound **1e** (Figure 1) to compare the biological activity values of the studied compounds bearing different structural motifs in their molecules. The authors developed and investigated structures similar to those reported earlier as important cytotoxic agents (Figure 1).^13−15^ The target structural architecture was again used as the key structural motif in their following studies presenting a synthesis of a large series of purine analogues containing either substituted piperazine or phenylcyclopentane as the additional structural motifs.^8^ The *in vitro* anticancer activity of all prepared compounds in several human cancer cell lines was studied. Several compounds of this series (**1f**–**1q**; Figure 1) displaying IC_50_ values lower than 10 μM were selected for a more detailed investigation in an enlarged panel of liver cancer cell lines. The experiments revealed that compound **1j** (R_1_ = H) induced apoptosis *in vitro* due to its high cytotoxic potential (IC_50_ < 5 μM; Table 1). The authors presented that the compound **1j** displayed a significant selectivity against anaplastic lymphoma kinase (Alk) and Bruton’s tyrosine kinase (BTK) over other kinases.^8^ The most successful compounds of this series (**1j**, **1l**, **1m**, **1n**, and **1q**; Figure 1) complexed with Alk, BTK, and DDR2 (discoidin domain-containing receptor 2). Their binding site interactions and binding affinities were analyzed by molecular docking and molecular dynamics simulations. The compounds **1j** and **1q** displayed similar interactions with the activation loop of the kinases. However, the investigation revealed that only compound **1j** reached the active sites of the kinases. The cell cycle and signaling pathway analyses exhibited that compound **1j** decreased phospho-SRC, phospho-Rb, cyclin E, and Cdk2 levels in liver cancer cells and induced apoptosis.^8^ Fludarabine, the 5-*O*-phosphorylated β-d-arabinofuranosyl derivative of 6-amino-2-fluoropurine, a medicinally used chemotherapeutic agent, was used as the positive reference compound (Table 1).^8^

**Table 1:** *In Vitro* Cytotoxicity (IC_50_ [μM] ± SD) of **1f**–**1q** in Three Human Cancer Cell Lines (Huh7, HCT116, and MCF7)^8^

compound	Huh7^a^	HCT116^b^	MCF7^c^
**1f**	9.0 ± 2.2	12.0 ± 1.5	5.2 ± 1.9
**1g**	34.4 ± 5.7	44.8 ± 9.5	10.5 ± 1.0
**1h**	8.0 ± 1.2	8.4 ± 1.5	6.5 ± 0.8
**1i**	7.1 ± 2.0	10.4 ± 1.4	8.6 ± 3.5
**1j**	1.0 ± 0.2	2.1 ± 0.3	0.1 ± 0.1
**1k**	1.4 ± 0.1	1.9 ± 0.6	6.4 ± 1.9
**1l**	3.8 ± 0.1	5.0 ± 2.1	1.0 ± 0.7
**1m**	1.6 ± 0.3	1.6 ± 0.5	9.1 ± 2.3
**1n**	0.1 ± 0.1	0.6 ± 0.3	4.1 ± 1.1
**1o**	0.04 ± 0.01	0.04 ± 0.03	0.16 ± 0.1
**1p**	6.3 ± 1.1	6.6 ± 0.4	12.6 ± 1.1
**1q**	1.2 ± 0.3	0.8 ± 0.1	2.8 ± 0.3
fludarabine	29.9 ± 20.0	8.3 ± 3.0	15.2 ± 0.1

a Huh7, hepatocyte derived liver carcinoma.

b HCT116, human colorectal carcinoma.

c MCF7, human breast adenocarcinoma.

**Figure 2:**
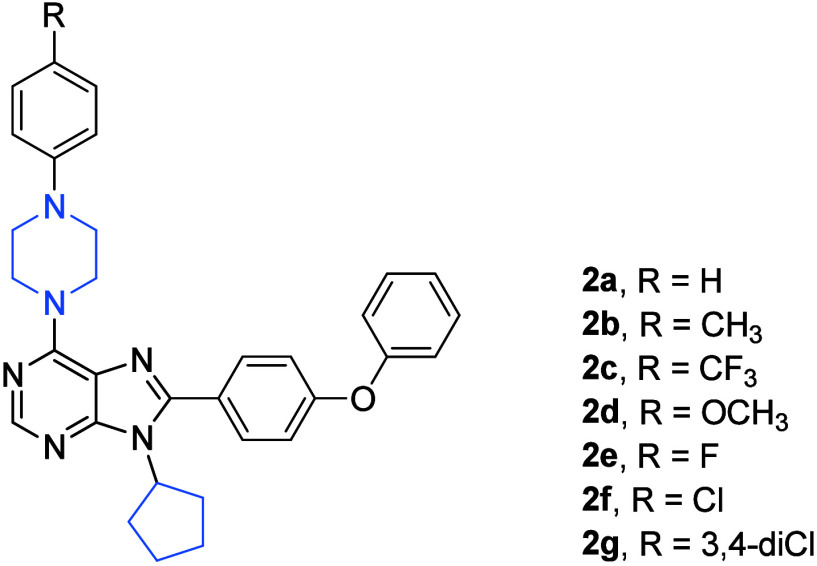
Structures of compounds **2a**–**2g**.

A subsequent study by the Turkish authors described a development and investigation of a series of 6-substituted-(phenylpiperazine)-8-(4-phenoxyphenyl)-9-cyclopentyl purine derivatives (**2a**–**2g**; Figure 2).^9^ The motivation for designing the novel 6,8,9-trisubstituted purine analogues was based on a general effort to focus on targeting the acquired resistance mechanisms in cancer cells. This resistance represents a significant difficulty in current medical methods for cancer treatment. The synthesis, starting from 4,6-dichloro-5-nitropyrimidine, involved a multistep process, resulting in a series of new targeted purine derivatives. Biological screening tests were performed using a sulforhodamine B (SRB) assay in human liver, colon, and breast cancer cells (Huh7, HCT116, and MCF7, respectively). Among the synthesized analogues, compounds **2a** and **2b** exhibited medium cytotoxic activity, using fludarabine as a positive reference compound, in terms of efficacy (Table 2). The disadvantage of the screening tests lies in the absence of tests made in nonmalignant cells. Nevertheless, this investigation pointed out the potential of purine derivatives with the phenyl group at the C(8) position as a scaffold for developing compounds with improved anticancer properties. The findings offered insights into the future exploration and development of novel agents in cancer research.

**Table 2:** Cytotoxicity Values (IC_50_ [μM] ± SD) of **2a**–**2g** in Three Cancer Cell Lines^9^

compound	Huh7^a^	HCT116^b^	MCF7^c^
**2a**	17.9 ± 0.9	17.2 ± 1.7	39.6 ± 4.8
**2b**	14.2 ± 1.4	13.7 ± 2.7	41.7 ± 3.8
**2c**	41.5 ± 8.3	21.8 ± 1.7	no inhibition
**2d**	23.6 ± 7.1	30.4 ± 4.0	no inhibition
**2e**	80.1 ± 16.0	19.5 ± 1.0	69.2 ± 11.8
**2f**	no inhibition	17.6 ± 5.3	no inhibition
**2g**	no inhibition	48.2 ± 9.6	no inhibition
fludarabine	29.9 ± 20.0	8.3 ± 3.0	15.2 ± 0.1

a Huh7, hepatocyte derived liver carcinoma.

b HCT116, human colorectal carcinoma.

c MCF7, human breast adenocarcinoma.

**Figure 3:**
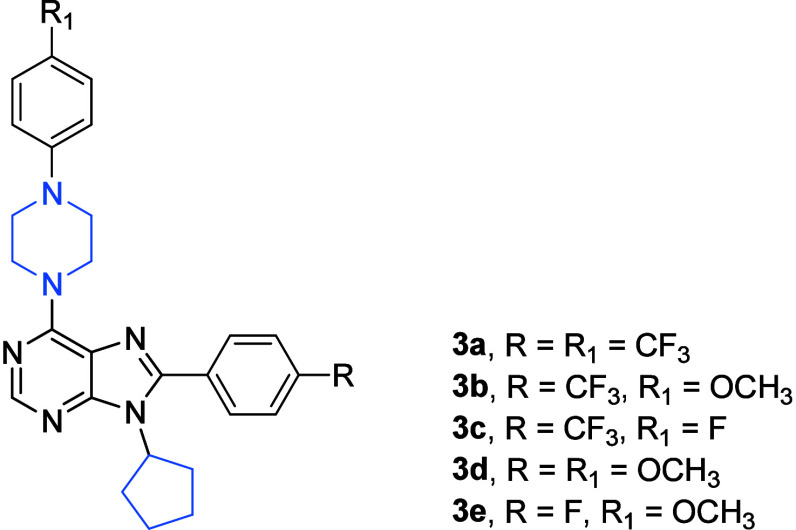
Structures of compounds **3a**–**3e**.

Finally, the third paper of the Turkish authors on this topic was focused both on making the series of the 6,8,9-trisubstituted purine analogues even broader and on increasing the number of the cancer cell lines involved in the screening tests.^10^ In this paper, a large series of 40 compounds, 6-substituted-(phenylpiperazine)-8-(4-substituted-phenyl)-9-cyclopentyl purines, were designed and synthesized by generally four-step synthetic processes that were described in detail.^10^ The reaction conditions were effectively optimized, and the final products were obtained with high purity and high yields in all synthetic steps.^10^ The *in vitro* cytotoxic effects of the synthesized target compounds were tested in the selected human cancer cell lines Huh7 (liver), HCT116 (colon), and MCF7 (breast), using an SRB assay.^10^ Among these analogues, compounds bearing 4-trifluoromethylphenyl (**3a**–**3c**; Figure 3), 4-methoxyphenyl (**3d**; Figure 3), and 4-fluorophenyl (**3e**; Figure 3) substituents at the C(8) center of the purine scaffold were the most potent and, therefore, analyzed both in the drug-resistant and the drug-sensitive hepatocellular cancer cell (HCC) panels as well. The compounds **3a** and **3d** displayed remarkable cytotoxic effects (IC_50_ = 2.9–9.3 μM) in Huh7, FOCUS, SNU475, SNU182, HepG2, and Hep3B cells, compared to fludarabine that was used again as a positive control (Table 3). The achieved results revealed that the studied compounds displayed favorable physicochemical characteristics for oral bioavailability and showed no toxicity end points, such as carcinogenicity, immunotoxicity, mutagenicity, or general toxicity.^10^

**Table 3:** *In Vitro* Cytotoxicity (IC_50_ [μM] ± SD) of Compounds **3a**–**3e** in Several Different Human Cancer Cell Lines (Huh7, HCT116, and MCF7) and in Drug-Sensitive Hepatocellular Carcinoma (HCC) Cell Lines (Huh7, Hep3B, HepG2, PLC, Mahlavu, FOCUS, SNU475, and SNU182)^10^

	cancer cell lines			HCC cancer cell lines							
comp.	Huh7	HCT116	MCF7	Huh7	Hep3B	HepG2	PLC	Mahlavu	FOCUS	SNU475	SNU182
**3a**	9.3 ± 1.1	10.8 ± 1.3	6.1 ± 0.7	9.3 ± 1.1	10.3 ± 1.0	6.6 ± 0.7	99.1 ± 19.8	19.8 ± 4.0	4.5 ± 0.2	17.0 ± 1.7	16.4 ± 0.8
**3b**	8.6 ± 1.4	24.0 ± 3.8	5.2 ± 0.8	8.6 ± 1.4	32.0 ± 6.4	5.1 ± 0.3	NI^a^	NI^a^	16.1 ± 1.6	54.8 ± 11.0	39.5 ± 4.0
**3c**	7.8 ± 0.9	15.7 ± 1.7	17.0 ± 1.9	7.8 ± 0.9	8.4 ± 0.7	5.9 ± 0.9	NI^a^	34.0 ± 2.7	10.7 ± 3.2	47.5 ± 3.8	81.9 ± 6.6
**3d**	2.9 ± 0.5	30.7 ± 4.9	47.7 ± 7.6	2.9 ± 0.5	17.8 ± 2.3	15.1 ± 1.2	NI^a^	41.7 ± 6.3	13.6 ± 2.0	53.3 ± 6.9	54.9 ± 8.2
**3e**	8.3 ± 1.0	13.0 ± 1.6	22.5 ± 2.7	8.3 ± 1.0	8.9 ± 0.4	6.7 ± 2.7	NI^a^	83.9 ± 16.8	11.2 ± 4.5	NI^a^	NI^a^
fludarab.^b^	29.9 ± 20.0	8.3 ± 3.0	15.2 ± 0.1	24.4 ± 4.9	27.8 ± 8.3	17.0 ± 3.4	41.7 ± 8.3	14.2 ± 1.4	13.7 ± 2.7	41.5 ± 12.5	37.2 ± 3.7

a NI = no inhibition.

b Fludarabine.

**Figure 4:**
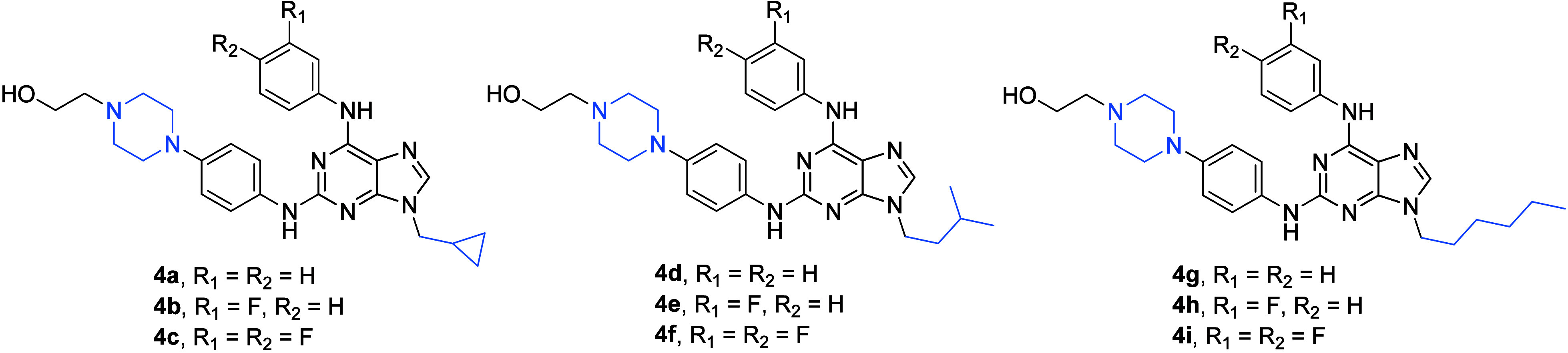
Structures of compounds **4a**–**4i**.

The structural motif of piperazine for the modification of the purine scaffold, combined with the alkyl cyclopropyl substituent of the *N*(9)-heteroatom, was applied quite often by various authors.^16^ A series of new 2,6,9-trisubstituted purines, the structures of which were designed on the basis of the previously developed Bcr-Abl inhibitors by the same authors, were recently synthesized and studied.^16,17^ Bcr-Abl is an oncoprotein with aberrant tyrosine kinase activity involved in the progression of chronic myeloid leukemia (CML), and it has been targeted by the inhibitors imatinib and nilotinib. A considerable number of 20–30% of patients who were treated by imatinib showed acquired or intrinsic resistance to the treatment during their disease. Therefore, their resistance to the drug has remained a considerable obstacle and a clinical challenge.^16^ Two types of the basic mechanism of resistance, either a Bcr-Abl-dependent or Bcr-Abl-independent one, were described.^16^ It follows from the above results that Bcr-Abl has remained a highly attractive target for designing and developing selective inhibitors capable of representing a novel class of potent therapeutic agents in treating leukemia.^16^ The series of the investigated compounds was rather broad,^16^ and therefore, only the most important compounds (**4a**–**4i**) are shown in Figure 4. Among them, **4b** should be highlighted on the basis of the structure–activity analysis for its potency against Bcr-Abl (IC_50_ = 0.015 μM) that was higher than that of the commercially used drugs imatinib and nilotinib.^16^ The compound **4b** displayed the most potent antiproliferative characteristics in three CML cells causing Bcr-Abl rearrangement (**4b**: IC_50_ = 0.015 ± 0.010 μM; Table 4). In addition, these purine-based compounds inhibited the growth of KCL22 cell lines expressing Bcr-AblT315I, Bcr-AblE255 K, and Bcr-AblY253H point mutants at micromolar concentrations. The commercial drugs imatinib and nilotinib were ineffective in inhibiting the growth of the KCL22 cells compared to **4a**–**4i** (Table 4). The molecular docking studies explained the structure–activity relationships of these purines in Bcr-AblWT and Bcr-AblT315I.^16^ Finally, the cell cycle cytometry assays and immunodetection showed that **4b** arrested the cells in the G1 phase and downregulated the protein levels downstream of Bcr-Abl in these cells.^16^

**Table 4:** Inhibition of the Recombinant Abl1 Kinase *In Vitro* by Several Successful Compounds (**4a**–**4i**) from the Investigated Series^16^

compound	IC_50_ [μM] ± SD
**4a**	0.037 ± 0.012
**4b**	0.015 ± 0.010
**4c**	0.020 ± 0.001
**4d**	1.240 ± 0.220
**4e**	0.131 ± 0.071
**4f**	1.760 ± 0.740
**4g**	0.834 ± 0.447
**4h**	0.674 ± 0.069
**4i**	1.680 ± 0.010

**Figure 5:**
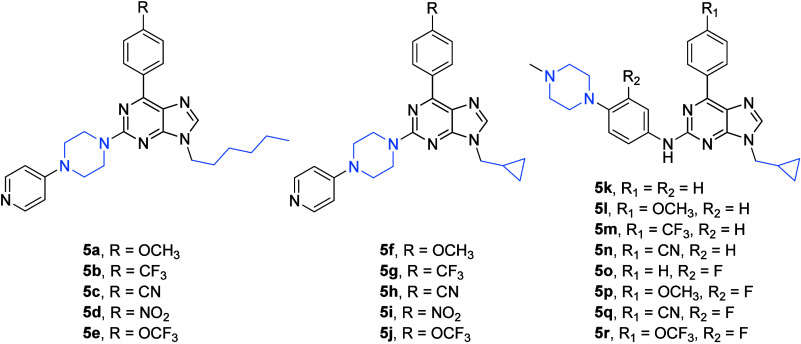
Structures of compounds **5a**–**5r**.

A combination of the *N*-arylpiperazine and alkyl cyclopropane structural motifs also appeared in another paper, published in the same period.^18^ In that investigation, the authors focused on a previous statement concerning aberrant activation of the Hedgehog (Hh) signaling pathway. It was found to be associated with the development and progression of pancreatic cancer.^19^ For that reason, blocking the Hh pathway was considered by using convenient inhibitors targeting the G protein coupled receptor Smoothened (SMO), a therapeutic target for the treatment of pancreatic cancer. In a previous paper presented by the same team,^20^ a new SMO ligand based on the purine scaffold (**5e**; Figure 5) was designed, and it showed high cytotoxicity in several cancer cell lines. In the following and most recent paper,^18^ the authors reported the design and synthesis of a broad series of new purine derivatives **5a**–**5r** (Figure 5) inspired by the structure of the pioneer compound **5e** (Figure 5). Some of the compounds of this broad series showed a high cytotoxic effect on Mia-PaCa-2, an Hh-dependent pancreatic cancer cell line, and low toxicity on non-neoplastic HEK293 cells compared with the effect of gemcitabine. This finding was documented by the compounds **5p** (IC_50_ = 4.56 μM), **5q** (IC_50_ = 4.11 μM), and **5r** (IC_50_ = 3.08 μM), whose cytotoxicity values were comparable with that of the pioneer structure **5e** (Figure 5; Table 5). Two of these purine derivatives also showed their ability to bind to SMO through NanoBRET assays (p*Ki* = 5.17 for **5p** and p*Ki* = 5.01 for **5r**, respectively), with higher affinities to **5e** (p*Ki* = 1.51). In addition, docking studies provided an insight into the purine substitution patterns related to the affinity in SMO. Finally, studies of the Hh inhibition by the selected purines, using a transcriptional functional assay based on the luciferase activity in the NIH3T3 Shh-Light II cells, demonstrated that **5q** reduced the GLI activity (IC_50_ = 6.4 μM) as well as diminished the expression of the Hh target genes in two specific Hh-dependent cell models, Med1 cells and mouse embryonic fibroblasts. Therefore, these results provided a basis for a possible design of next generation SMO ligands that could become potentially selective cytotoxic agents for treating pancreatic cancer.^18^ Gemcitabine, another clinically used chemotherapeutic agent, was used as a positive reference compound for comparing its effect with those displayed by the compounds of the studied series of purine derivatives (Table 5).

**Table 5:** *In Vitro* Cytotoxicity (IC_50_ [μM] ± SD) Values of **5a**–**5r** in Three Pancreatic Cancer Cell Lines and in Nonmalignant Cells HEK293^18^

compound	BxPC-3^a^	AsPC-1^a^	MIA-PaCa-2^a^	HEK293^b^
**5a**	7.13 ± 0.47	5.14 ± 1.39	3.96 ± 0.23	7.07 ± 3.95
**5b**	5.65 ± 0.86	5.37 ± 0.21	1.39 ± 0.09	4.69 ± 1.82
**5c**	4.48 ± 0.36	4.68 ± 0.74	1.56 ± 0.02	41.18 ± 3.84
**5d**	10.6 ± 1.82	5.24 ± 2.37	9.21 ± 0.37	3.93 ± 1.65
**5e**^c^	4.10 ± 0.90	1.70 ± 0.05	10.0 ± 0.07	>50
**5f**	>25	9.66 ± 3.52	4.55 ± 0.50	8.98 ± 5.95
**5g**	5.07 ± 0.16	5.75 ± 0.22	2.96 ± 0.11	2.99 ± 3.07
**5h**	>25	>25	>25	>50
**5i**	>25	2.31 ± 0.23	>25	1.33 ± 1.74
**5j**	3.28 ± 0.11	5.28 ± 0.49	2.88 ± 0.02	3.76 ± 1.57
**5k**	>25	>25	>25	>50
**5l**	>25	4.35 ± 4.73	2.97 ± 1.32	11.88 ± 2.06
**5m**	5.12 ± 0.07	6.85 ± 0.38	1.76 ± 0.58	5.37 ± 3.97
**5n**	>25	5.35 ± 3.27	>25	61.10 ± 4.08
**5o**	>25	>25	8.30 ± 0.17	>50
**5p**	10.1 ± 0.79	>25	4.56 ± 0.16	41.16 ± 6.27
**5q**	>25	3.95 ± 1.63	4.11 ± 1.12	>50
**5r**	5.80 ± 0.07	9.56 ± 1.60	3.08 ± 0.29	10.93 ± 5.57
gemcitabine	12.12 ± 1.67	1.33 ± 0.29	13.45 ± 1.33	29.62 ± 1.44

a BxPC-3, AsPC-1, and MIA-PaCa-2, pancreatic carcinoma cell lines.

b HEK293, human embryonic kidney cells.

c Compound **5e** appeared already in ref (20) as the pioneer compound of the series **5a**–**5r**.

## 3 Purine Derivatives Mimicking Cytokinins

**Figure 6:**
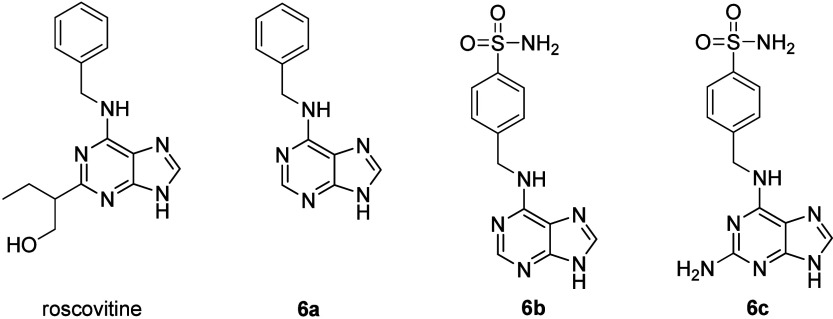
Structures of roscovitine and compounds **6a**–**6c**.

Cyclin-dependent kinases (CDKs) are recognized as the primary regulators of the cell cycle and are overexpressed in various types of cancer. Indian authors applied this knowledge during the investigation of *N*^6^-benzylaminopurines and their sulfonamide derivatives, which represents another way of structural modification of the purine scaffold, yielding CDK inhibitors.^21^ It has been found that inhibiting CDKs with small molecules can reduce tumor growth and thus benefit cancer patients. In the investigation performed by the authors,^21^ one of the potentially cytotoxic compounds (**6a**; Figure 6) was designed without the presence of the sulfonamide motif, while two additional compounds (**6b** and **6c**; Figure 6) represented sulfonamide-decorated *N*^6^-benzylaminopurines, i.e., α-(purin-6-ylamino)-*p*-toluenesulfonamide (**6b**; Figure 6) and α-(2-aminopurin-6-ylamino)-*p*-toluenesulfonamide (**6c**; Figure 6). All of these compounds (**6a**–**6c**) were synthesized to study their potential cytotoxic effects. Compound **6a** was known as a plant cytokinin, a group of plant hormones, known also as plant growth regulators.^22,23^ The Indian authors^21^ showed crystallographic analysis of the studied compounds, resulting in a finding that **6b** crystallized in *P*1̅ of the triclinic system, while **6c** crystallized in *C*2/*c* or *P*2_1_/*c* of the monoclinic systems. The Hirshfeld surface and X-ray crystallographic studies discovered that **6c** possessed a stronger noncovalent interaction ability than roscovitine, a known CDK inhibitor. The *in silico* analysis showed that **6c** had a higher binding affinity for the ATP binding sites of the CDK1, CDK2, and CDK4 receptors than roscovitine. The enhanced binding affinity of **6c** with the CDKs was associated with strong noncovalent interactions between **6c** and the specific amino acids in the CDKs. Cytotoxicity studies were conducted on a glioblastoma cell line (U251) by incubating cells with **6a**–**6c**, roscovitine (Figure 6), and the established anticancer drug temozolomide (TMZ). Compound **6c** (IC_50_ = 66.12 ± 1.09 μM) demonstrated better cytotoxicity than compound **6b** (IC_50_ = 81.22 ± 0.30 μM), roscovitine (IC_50_ = 127.10 ± 0.47 μM), and TMZ (IC_50_ = 165.11 ± 1.00 μM). It was already known that CDK inhibition leads to cell cycle arrest. Interestingly, the authors found that **6c** induced the G2/M phase cell cycle arrest by increasing the percentage of the G2/M phase cell population from 21.08% to 47.79% in the U251 cells.^21^ Based on these results, **6c** showed an anticancer effect by binding to the ATP binding site of the CDKs. Therefore, **6c** was appointed as a potential lead molecule in the future development of effective anticancer agents.^21^ However, a general structure–activity relationship analysis of compounds **6a**–**6c** revealed that their cytotoxicity was rather low in comparison with other structures mentioned in this review.

The findings of the inactivity of **6a** and the relatively low cytotoxicity of **6b** and **6c** were in accordance with the results of the earlier investigations of Czech authors, who searched for the cytotoxicity and other types of biological effects of cytokinins for a potential augmenting of human health.^24,25^ Even if cytokinins are compounds bearing the purine scaffold in their molecules as well, the so far performed investigations resulted in findings that these plant products display effects in plant growth regulation and show no ability to develop into medicinally important plant products.^26^ In turn, nobody has yet investigated the effect of compounds **6b** and **6c** as inhibitors of cytokinin oxidase/dehydrogenase.

## 4 A Series of Thiazepinopurines

**Figure 7:**
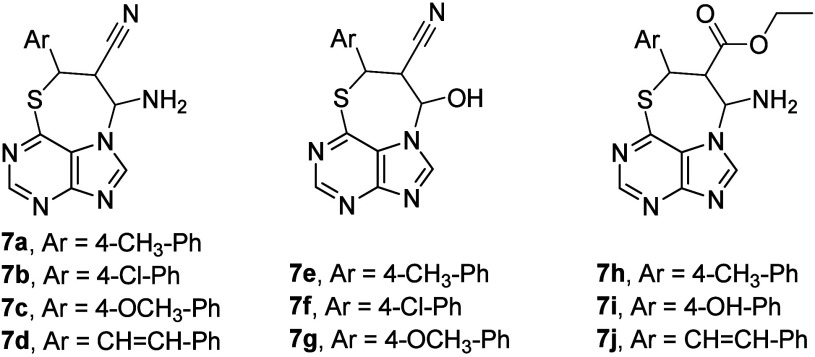
Structures of compounds **7a**–**7j**.

Novel thiazepinopurines comprising three different but similar general structures (Figure 7) were designed and synthesized by means of adopting the molecular overlay approach, and they were expected to display a cytotoxicity effect and CDK2 inhibition potential.^27^ This synthetic strategy was based on the heteroannelation of purines with thiazepine. Cytotoxicity of the prepared thiazepinopurine derivatives was investigated in three different types of cancer cells (HepG2, MCF7, and PC-3), using normal cells (WI38) as reference cells in this investigation.^27^ Among the studied compounds (**7a**–**7j**), two of them (**7b** and **7c**) exhibited significant antiproliferative activity in the tumor cells (Table 6). They showed cytotoxicity in the IC_50_ range of 5.52–17.09 μM in comparison with roscovitine, used as a positive reference compound (IC_50_ = 9.32–13.82 μM). In addition, both successful compounds (**7b** and **7c**) displayed acceptable selectivity index values (SI = 3.00–7.15) in the tested cancer cells. The 4-chlorophenyl analogue **7b** showed the best selectivity index, and hence, it was subjected to an additional investigation to determine its proper biological effects. Accordingly, the CDK2 inhibition potential, the induction of apoptosis, and the cell cycle analysis in the MCF7 cancer cells were evaluated. The results revealed that **7b** displayed a potent CDK2 inhibition potential with IC_50_ = 0.219 μM.^27^ The findings also showed that **7b** arrested the MCF7 cell cycle at the S phase, together with apoptosis induction, by the increased expression of BAX, Caspase-8, and Caspase-9 markers and with the concomitant decrease in Bcl-2 expression.^27^ Besides, the probable interaction of **7b** with the CDK2 binding pocket was investigated by molecular docking.^27^Table 6 summarizes the *in vitro* antiproliferative effects of the compounds **7a**–**7j**, compared with those of doxorubicine and roscovitine, both serving as positive reference compounds.

**Table 6:** *In Vitro* Antiproliferative Effect (IC_50_ [μM] ± SD) of the Synthesized Compounds **7a**–**7j**^27^

compound	HepG2^a^	MCF7^b^	PC-3^c^
**7a**	50.81 ± 2.9	68.47 ± 3.5	58.75 ± 3.3
**7b**	8.06 ± 0.7	5.52 ± 0.3	13.15 ± 0.9
**7c**	15.63 ± 1.3	9.27 ± 0.7	17.09 ± 1.3
**7d**	62.53 ± 3.4	74.58 ± 3.7	80.36 ± 3.9
**7e**	43.42 ± 2.6	26.53 ± 1.9	29.70 ± 1.9
**7f**	38.25 ± 2.4	42.73 ± 2.5	48.68 ± 2.7
**7g**	47.62 ± 2.5	27.42 ± 1.8	64.31 ± 3.6
**7h**	77.44 ± 3.8	83.25 ± 4.2	93.13 ± 4.8
**7i**	28.89 ± 2.0	16.19 ± 1.3	45.96 ± 2.3
**7j**	57.30 ± 3.2	61.73 ± 3.3	78.40 ± 3.7
doxorubicine	4.50 ± 0.2	5.23 ± 0.31	4.17 ± 0.2
roscovitine	13.82 ± 1.15	12.24 ± 1.17	9.32 ± 0.49

a HepG2, hepatocyte carcinoma.

b MCF7, breast adenocarcinoma.

c PC-3, prostate adenocarcinoma.

## 5 Purine Derivatives in Nanovesicles with the Ability of Coordinating Metal Ions

**Figure 8:**
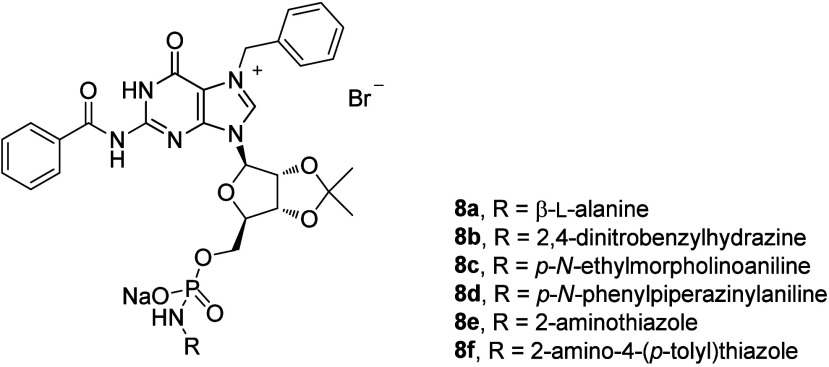
Structures of compounds **8a**–**8f**.

Translation of mRNA is one of the processes adopted by cancer cells to maintain survival via phosphorylated eukaryotic initiation factor 4F (eIF4F) overexpression.^28^ It consists of the ATP-dependent RNA helicase eIF4A, the large scaffolding protein eIF4G, and the 5′-terminus of mRNA cap-binding subunit eIF4E. The recognition of the 7-methylguanosine nucleoside triphosphate cap at the 5′-terminus of mRNA by eIF4E is essential for initiating the cap-dependent translation. Dysregulation of the cap-dependent translation is linked to the development and progression of cancer.^28^ Once the phosphorylated subunit eIF4E, further identified as (*p*)-eIF4E, binds to the cap structure of mRNA, it supports a nonstop translation process.^28^ In this regard, a series of the new GMP analogues were synthesized to target eIF4E and suppress its binding to the mRNA cap structure (**8a**–**8f**; Figure 8).^28^ The compounds of this series were tested in three types of cancer cell lines: Caco-2, HepG2, MCF7, and normal kidney cells (Vero cells) (Table 7). Most of the compounds showed a high potency in breast cancer cells (MCF7), characterized by the highest cancer type for overexpression of (*p*)-eIF4E. Compound **8b** was found to be the most active in three cancer cell lines, colon (Caco-2; IC_50_ = 31.40 ± 0.53 μM), hepatic (HepG2; IC_50_ = 27.15 ± 2.23 μM), and breast (MCF7; IC_50_ = 21.71 ± 2.24 μM), respectively, while it was nontoxic in the Vero cells (IC_50_ > 100 μM; Table 7). To enhance the cytotoxicity of the most successful compound **8b**, chitosan-coated niosomes loaded with the compound **8b** (Cs/**8b**-NSs) were developed (as kinetically enhanced molecules) to improve the anticancer effect of **8b** through nanoassembly.

**Table 7:** Cytotoxicity (IC_50_ [μM] ± SD) of **8b** and Its Nanoassembly (Cs/**8b**-NSs) in Three Cancer Cell Lines and in Nonmalignant Vero Cells^28^

compound	Caco-2^a^	HepG2^b^	MCF7^c^	Vero^d^
**8b**	31.40 ± 0.53	27.15 ± 2.23	21.71 ± 2.24	>100
Cs/**8b**-NSs	16.15 ± 0.66	26.66 ± 1.18	6.9 ± 0.86	>100
rapamycin	27.68 ± 1.63	47.55 ± 3.83	5.62 ± 0.24	>100
ribavirin	9.78 ± 0.94	63.94 ± 3.43	10.21 ± 0.15	>100

a Caco-2, colon adenocarcinoma.

b HepG2, hepatocyte carcinoma.

c MCF7, breast adenocarcinoma.

d Vero, nonmalignant African green monkey kidney cells.

Current chemotherapeutics delivery approaches include various nanocarriers, including metallic nanoparticles, polymeric nanoparticles, macromolecules, silica nanoparticles, and nanovesicles, as mentioned in the original paper.^28^ Nanovesicles (liposomes and niosomes) are lipid-based nanocarriers that are reported to encapsulate natural and synthetic chemotherapeutics, enhancing their hydrophilicity and therapeutic effects. Liposomes are formed mainly of phospholipids (such as phosphatidylcholine and phosphatidylethanolamine) self-assembled in an aqueous medium, forming lipid bilayer nanovesicles. On the other hand, niosomes, i.e., nanocarriers composed of nonionic surfactants, have gained attention as reliable and modern alternative nanocarriers to liposomes.^28^ They are composed mainly of cholesterol and nonionic surfactants engineered by self-assembly in an aqueous phase, generating bilayer vesicles. Niosomes have attractive properties, making them promising nanovesicles in cancer therapy. They are stable, biocompatible, biodegradable, and safe carriers with minimal immunogenic effects. In addition, negatively charged niosomes could be coated with polycationic chitosan via an electrostatic interaction. Chitosan is a biocompatible and biodegradable natural polymer with mucoadhesive properties. This finding led to the adhesion of niosomes to the cancer cell membrane that prolonged the residence time of the niosomes at the site of action and achieved a controlled release of the burden at cancer cells.^29^ Thus, to exploit the benefits of loading anticancer drugs in chitosan-coated niosomes (Cs/NSs), **8b** was loaded into Cs/NSs, forming Cs/**8b**-NSs. Then, the designed niosomal formulation was characterized in terms of size, polydispersity index, surface charge, and shape.^28^ The efficiency of entrapment and the release behavior of the loaded **8b** out of the niosomal formulation were investigated as well.^28^

The prepared Cs/**8b**-NSs showed pronounced cytotoxicity compared to that of free **8b** in Caco-2 (IC_50_ = 16.15 ± 0.66 μM), HepG2 (IC_50_ = 26.66 ± 1.18 μM), and MCF7 (IC_50_ = 6.90 ± 0.86 μM), respectively (Table 7). The prepared Cs/**8b**-NSs was nontoxic in Vero cells (IC_50_ > 100 μM). Then, the expression of both phosphorylated and nonphosphorylated Western blot techniques was conducted in MCF7 cells treated with the most active compounds (based on the obtained IC_50_ values) to determine the total protein expression of both eIF4E and (*p*)-eIF4E. Interestingly, the most active compounds selected for this study displayed 35.8–40.7% inhibition of (*p*)-eIF4E expression when evaluated in the MCF7 cancer cells compared to ribavirin, used as a positive control.^28^ The chitosan-coated niosomal formulation (Cs/**8b**-NSs) has been proven to show the best inhibition (40.7%) within the given study.^28^ The findings of the *in silico* molecular docking, simulation dynamic studies, and experimental investigation suggested the potential application of niosomal nanovesicles as promising nanocarriers for the targeted delivery of the newly synthesized compound **8b** to eIF4E. These outcomes supported a possible application of Cs/**8b**-NSs in targeted cancer therapy.^28^

**Figure 9:**
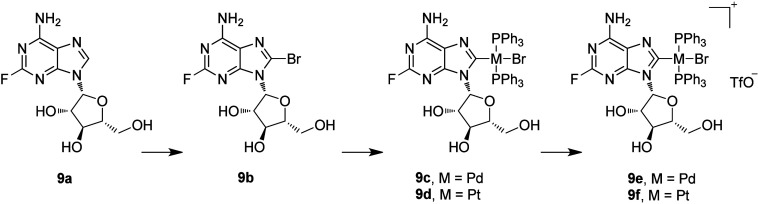
Scheme of the synthesis of fludarabine complexes **9c**–**9f**, using fludarabine (**9a**) as a source molecule.

The purine derivative fludarabine (**9a**; Figure 9) has been a part of a frontline therapy for chronic lymphocytic leukemia (CLL) for some time.^30^ It showed positive effects on solid tumors, such as melanoma, breast, and colon carcinoma, in clinical phase I studies. The treatment of CLL cells with combinations of fludarabine (**9a**) and metal complexes of antitumor natural products, e.g., illudin-M ferrocene, has led to synergistically enhanced apoptosis.^30^ The subsequent research study by a German team focused on developing different complexes of fludarabine (**9c**–**9f**; Figure 9), in which compound **9b** (Figure 9) served as a key intermediate in the synthesis.^30^ Four complexes (**9c**–**9f**) bearing a *trans*-[Br(PPh_3_)_2_]Pt/Pd fragment bound to the C(8) atom via formal η^1^-sigma or η^2^-carbene bonds were synthesized in two or three steps without protecting polar groups on the arabinose skeleton or on the adenine scaffold (Figure 9).^30^ The platinum complexes were more cytotoxic than their palladium analogues, with low single-digit micromolar IC_50_ values in the cells of various solid tumor entities, including the cisplatin-resistant ones, and certain B-cell lymphoma and CLL, presumably due to the 10-fold higher cellular uptake of the platinum complexes.^30^ However, the palladium complexes interacted more readily with the isolated calf thymus DNA.^30^ Interestingly, the platinum complexes showed vastly greater selectivity for cancer over nonmalignant cells when compared with fludarabine.^30^Table 8 summarized the cytotoxicity of **9c**–**9f** in several cancer cell lines and in comparison with the cytotoxicity of fludarabine (**9a**).^30^

**Table 8:** Results (IC_50_ [μM] ± SD)^a^ of the SRB Cytotoxicity Assay Applied to **9a** and **9c**–**9f** after 72 h of Treatment^30^

compound	A2780^b^	A2780cis^c^	A549^d^	MCF7^e^	HT29^f^	CCD18Co^g^
**9a**	0.17 ± 0.05	0.24 ± 0.00	0.13 ± 0.04	0.24 ± 0.06	1.8 ± 0.57	0.62 ± 0.44
**9c**	3.18 ± 1.45	6.55 ± 2.4	7.74 ± 2.90	17.64 ± 8.43	23.85 ± 9.60	>30
**9d**	1.06 ± 0.22	1.63 ± 0.2	1.77 ± 0.4	1.10 ± 0.27	4.07 ± 0.83	>30
**9e**	3.99 ± 2.28	7.67 ± 1.70	9.83 ± 5.42	17.23 ± 8.42	23.05 ± 8.18	>30
**9f**	0.97 ± 0.23	1.56 ± 0.32	1.50 ± 0.3	1.17 ± 0.27	3.32 ± 1.18	>30

a An average value of three independent experiments.^30^

b A2780, human ovarian carcinoma.

c A2780cis, resistant derivative of A2780.

d A549, human lung carcinoma.

e MCF7, human breast carcinoma.

f HT29, colorectal carcinoma.

g CCD18Co, nonmalignant human fibroblasts.

Alkaline earth metals have a considerably greater tendency to form complexes compared to alkali metals due to their smaller ionic radii and higher positive charge density. The significance of a complex formation is particularly pronounced for smaller cations, e.g., Mg^2+^ and Ca^2+^, which explains why the octahedral [M(H_2_O)_6_]^2+^ ion is commonly found in aqueous solutions.^31^ It was observed that the coordination numbers increase from Mg^2+^ to Ba^2+^.^31^ Large metal ions, like Sr^2+^ and Ba^2+^, can have varying coordination values, ranging from 3 to 12, with the most common ones being 7, 8 and 9.^31,32^

For that reason, alkaline earth metal (Mg^2+^, Ca^2+^, Sr^2+^, and Ba^2+^) complexes of guanine (Gu), the nucleobase bearing the purine scaffold, were synthesized, and their potential cytotoxic effects were investigated.^32^ The full characterization of these complexes by several relevant analytical methods demonstrated that the structures of the metal complexes of guanine were well assigned.^32^ The molecular formulas of the complexes were proposed on the basis of elemental analysis as [Mg(Gu)_2_Cl_2_]·4H_2_O, [Ca(Gu)_2_Cl_2_]·3H_2_O, [Sr(Gu)_3_Cl_2_]·5H_2_O, and [Ba(Gu)_3_Cl_2_](Gu)·5H_2_O.^32^ The molar conductance measurement showed that the complexes were nonelectrolytic.^32^ The mode of chelation of guanine through the N(7) and O(6) sites was proven and explained using FTIR and ^1^H NMR spectral analysis.^32^ Thermal analysis in a nitrogen atmosphere showed that the complexes were stable up to 100 °C. Their decomposition started above this temperature.^32^ The crystal size and the dislocation density of the complexes were determined using XRD data.^32^ Finally, guanine and its complexes were subjected to cytotoxicity tests in the HeLa cancer cell line and to antimicrobial and antifungal screening tests in five G^+^, eight G^–^ microorganisms, and three fungi. Guanine itself exhibited no antibacterial activity; however, its metal complexes showed significant antimicrobial and antifungal effects with no selectivity among the tested microorganisms.^32^ The *in vitro* cytotoxicity testing revealed that the alkaline earth metal complexes of guanine exhibited potential cytotoxic activity, showing LC_50_ = 18.55–40.61 μg·mL^–1^. Thus, each of the alkaline earth metal complexes of guanine demonstrated cytotoxicity in the HeLa cell line.^32^ However, no guanine–metal complex displayed higher cytotoxicity than cisplatin, a pharmacologically used agent for cancer treatment. The results of this investigation revealed that a convenient metal ion selection, to be coordinated in a biologically active compound, may proceed with the preparation of novel types of potential agents for PET screening with a simultaneous cytotoxic effect.^33^

**Figure 10:**
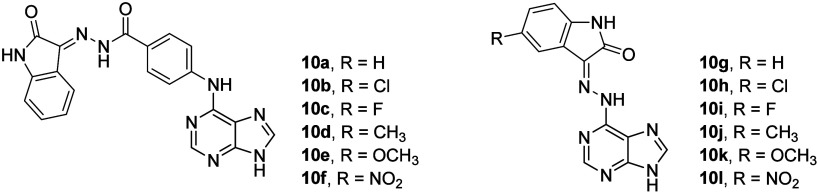
Structures of compounds **10a**–**10l**.

It was shown in papers dealing with cytotoxicity studies that the traditional single-treatment strategy for cancer is frequently unsuccessful due to the complexity of cellular signaling.^34^ However, the suppression of multiple targets has been vital to defeat tumor cells. For that reason, novel hybrid anticancer agents (conjugates) were developed for treating cancer more successfully.^34^ Such a strategy has been known for a long time and is used generally quite often, also in our team.^35−37^ Based on a molecular hybridization strategy, the required conjugates were designed, targeting multiple protein kinases in cancer cells.^34^ The studied hybrid agents combined purine and isatin (also known as tribulin; 1*H*-indole-2,3-dione) moieties in their structures together with 4-aminobenzohydrazide and hydrazine as different linkers. A series of compounds (**10a**–**10l**; Figure 10) were synthesized, and their biological effects were studied.^34^ Having those two moieties in one molecule enabled the capability of inhibiting multiple kinases, such as human epidermal receptor (EGFR), human epidermal growth factor receptor 2 (HER2), vascular endothelial growth factor receptor 2 (VEGFR2), or cyclin-dependent kinase 2 (CDK2).^34^ The cytotoxicity effect was evaluated by performing cytotoxicity and kinase inhibition assays, cell cycle analysis, and BAX, Bcl-2, Caspase-3, and Caspase-9 protein level determination assays.^34^ The results showed that the studied hybrids treated the cancer by inhibiting both cell proliferation and metastasis.^34^ A molecular docking study was performed to predict possible binding interactions in the active site of the investigated protein kinase enzymes.^34^ The most successful compound **10k** (Figure 10) from this series was docked in the active sites of the kinase proteins EGFR, VEGFR2, and Her2 to investigate its biological activity and to predict possible types of drug–receptor interactions.^34^ Erlotinib, sorafenib, and lapatinib were used as the reference compounds because they are the cocrystallizing ligands in the kinase proteins EGFR, VEGFR2, and Her2, respectively. The results of this study revealed that **10k** retained its activity in the nanomolar range in the inhibition of EGFR, VEGFR2, Her2, and CDK2, compared to the reference compounds (Figure 10, Table 9).^34^ In addition, the cell cycle analysis and the BAX, Bcl-2, Caspase-3, and Caspase-9 protein level determination assays indicated the apoptosis-inducing effect of **10k**. Overall, this reviewed work presented isatin–purine hybrid compounds as novel compounds targeting multiple kinases that may prove useful in the discovery of new cytotoxic therapeutics.^34^ Therefore, focusing on the experimental results, **10k** proved its potential as the most successful compound of this series of compounds. It showed the highest *in vitro* cytotoxicity in HepG2, MCF7, MDA-MB-231, and HeLa cancer cell lines (Table 9) and kinase inhibitory effects in EGFR, VEGFR2, Her2, and CDK2 comparable with those of the used reference compounds (Table 10).^34^

**Table 9:** Cytotoxicity Effect (IC_50_ [μM] ± SD) of Isatin-Purine Hybrid Compounds **10a**–**10l** in Four Cancer Cell Lines^34^

	*in vitro* cytotoxicity (IC_50_ [μM] ± SD)			
compound	HepG2^a^	MCF7^b^	HeLa^c^	MDA-MB-231^d^
**10a**	54.62 ± 3.1	47.26 ± 3.0	62.38 ± 3.4	59.67 ± 2.9
**10b**	88.14 ± 4.0	>100	>100	82.13 ± 3.8
**10c**	42.35 ± 2.7	22.31 ± 1.8	30.69 ± 2.3	19.05 ± 1.4
**10d**	60.38 ± 3.2	76.38 ± 3.7	>100	91.60 ± 4.5
**10e**	48.12 ± 2.9	40.57 ± 2.7	53.49 ± 3.2	29.78 ± 2.0
**10f**	75.56 ± 3.5	65.01 ± 3.4	84.57 ± 4.1	72.24 ± 3.4
**10g**	12.89 ± 1.0	15.60 ± 1.3	17.63 ± 1.4	24.83 ± 1.9
**10h**	26.45 ± 1.8	33.04 ± 2.3	46.72 ± 2.8	41.29 ± 2.5
**10i**	31.70 ± 2.1	38.82 ± 2.5	51.20 ± 3.0	45.21 ± 2.7
**10j**	18.06 ± 1.3	27.53 ± 2.1	43.51 ± 2.7	32.55 ± 2.2
**10k**	9.61 ± 0.8	10.78 ± 0.9	8.93 ± 0.8	14.89 ± 1.2
**10l**	39.73 ± 2.5	58.32 ± 3.2	69.50 ± 3.6	67.42 ± 3.2
sunitinib	6.82 ± 0.5	5.19 ± 0.4	7.48 ± 0.6	8.41 ± 0.7

a HepG2, hepatocyte carcinoma.

b MCF7, breast adenocarcinoma.

c HeLa, cervical cancer.

d MDA-MB-231, breast carcinoma.

**Table 10:** Kinase Inhibitory Effects of **10k** in the Protein Kinases EGFR, Her2, VEGFR2, and CDK2^34^

compound	kinase protein	IC_50_ [μM]
**10k**	CDK2	0.534
roscovitine	CDK2	0.143
**10k**	EGFR	0.143
erlotinib	EGFR	0.041
**10k**	Her2	0.150
lapatinib	Her2	0.051
**10k**	VEGFR2	0.192
sorafenib	VEGFR2	0.049

## 6 Purine Derivatives Bearing 1,4-Disubstituted 1,2,3-Triazole System

**Figure 11:**
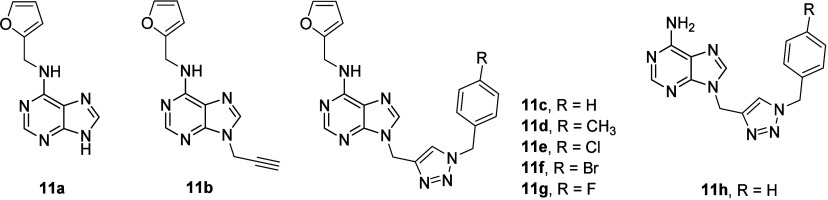
Structures of compounds **11a**–**11h**.

The cytotoxicity of two series of new 1,2,3-triazolylpurine derivatives was investigated.^38^ The authors^38^ used the term hybrid to describe the prepared compounds containing a 1,4-disubstituted 1,2,3-triazole system. However, we prefer not to use this term for describing the compounds **11a**–**11h** (Figure 11) because the 1,4-disubstituted 1,2,3-triazole system represents the isosteric mimic of the *trans*-amide bond in terms of the biological activity of the studied compound, and therefore, the compounds **11a**–**11h** should not be described as hybrid molecules or conjugates.^39^ These compounds bearing the 1,4-disubstituted 1,2,3-triazole system in their molecules were synthesized in considerable yields (up to 87%), starting from benzyl azides and purine-alkynes, by the Cu(I)-catalyzed 1,3-dipolar cycloaddition reaction (click reaction).^38^ The developed series of compounds employed kinetin (another cytokinin, plant hormone; cf., Section 3) and adenine as precursors, respectively. The *in vitro* antiproliferative activity of all synthesized compounds was tested in two breast cancer cell lines (MCF7 and MDA-MB-231) and in a nontumor cell line (MCF-10A) to evaluate their cytotoxicity, toxicity, and selectivity.^38^ Eight compounds (**11a**–**11h**; Figure 11) of about 20 synthesized compounds were active in both tested tumor cell lines, especially the kinetin derivatives, which displayed better results than the adenine derivatives. The original kinetin molecule (**11a**; Figure 11, Table 11) was not active (again in agreement with the above cited findings; cf. Section 3), suggesting that the structural changes in its derivatives were favorable to induce cytotoxic effects in the tested cells.^38^ The compounds **11e** and **11f** were the most active ones of the investigated series, displaying IC_50_ = 22.3 μM and IC_50_ = 22.9 μM for MCF7 and IC_50_ = 9.3 μM and IC_50_ = 16.7 μM for MDA-MB-231, respectively (Table 11). However, these compounds showed toxicity in nontumor cells that was comparable with their cytotoxicity in the tumor cell lines. Doxorubicine was used as a positive reference compound.^38^ Therefore, based on the structure–activity relationship analysis, this series of compounds was rather unsuccessful due to the comparable cytotoxicity values found in the malignant and nonmalignant cells.

**Table 11:** Antiproliferative Activity of Kinetin (**11a**), Its Derivatives **11b**–**11g**, and Adenine Derivative **11h** Obtained by MTT Assay for Tumor Cell Lines (MCF7 and MDA-MB-231) and Nonmalignant Cells (MCF-10A), Using Doxorubicine as a Reference Compound^38^

	antiproliferative activity (IC_50_ [μM] ± SD)		
compound	MCF7^a^	MDA-MB-231^a^	MCF-10A^b^
**11a**	>100	>100	>100
**11b**	76.6 ± 6.7	99.0 ± 1.5	90.9 ± 5.7
**11c**	45.8 ± 1.2	24.0 ± 0.9	28.3 ± 0.8
**11d**	37.0 ± 0.3	47.3 ± 3.3	27.5 ± 2.2
**11e**	22.3 ± 2.9	9.3 ± 1.7	16.4 ± 0.6
**11f**	22.9 ± 1.6	16.7 ± 1.8	24.6 ± 0.8
**11g**	69.6 ± 1.5	38.1 ± 5.7	23.9 ± 1.4
**11h**	94.4 ± 4.0	>100	>100
doxorubicine	0.7 ± 0.1	0.9 ± 0.1	0.6 ± 0.2

a MCF7 and MDA-MB-231, breast adenocarcinoma cell lines.

b MCF-10A, nonmalignant human epithelial cells.

**Figure 12:**
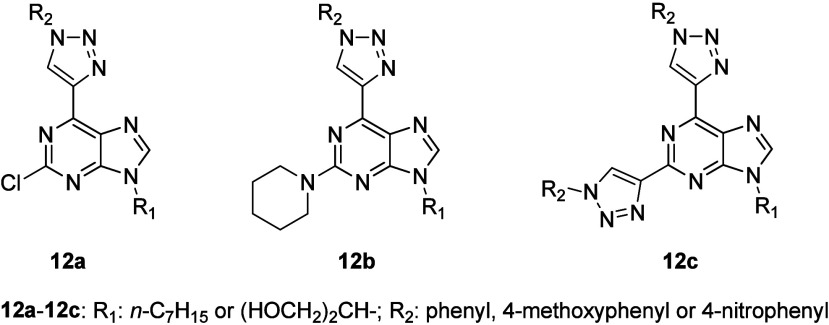
General structures of **12a**–**12c**.

Despite the low success of the above-described series of compounds shown in Figure 11,^38^ 1,4-disubstituted 1,2,3-triazolylpurine compounds still represent a considerable way of structural modification of the target biologically active scaffold.^40^ The application of the 1,2,3-triazole motif in developing novel biologically active compounds showed increasing importance in general.^41,42^ A novel design toward the C–C bonded 2,6-bis(1*H*-1,2,3-triazol-4-yl)-9*H*-purine and 2-piperidinyl-6-(1*H*-1,2,3-triazol-4-yl)-9*H*-purine derivatives was established using a combination of the Mitsunobu and Sonogashira reactions, the Cu(I)-catalyzed azide–alkyne cycloaddition (click reaction), and the S_N_Ar reaction.^40^ Their general structures **12a**–**12c** are shown in Figure 12, in which the most successful substituents R_1_ and R_2_, appearing in structures **12a**–**12c**, are identified. Eleven examples of 2,6-bis-(1,2,3-triazolyl)purine and 14 examples of 2-piperidinyl-6-(1,2,3-triazolyl)purine intermediates were synthesized, in 38–86% and 41–89% yields, respectively.^40^ The prepared 1,4-disubstituted 1,2,3-triazolylpurine compounds expressed good fluorescent properties, which were studied both in solution and in the thin layer film for the first time.^40^ Quantum yields reached 49% in DMSO for the bis-(1,2,3-triazolyl)purines and 81% in DCM and 95% in DMSO for the mono-(1,2,3-triazolyl)purines. The performed biological studies in the mouse embryo fibroblast, the human keratinocyte, and the transgenic adenocarcinoma of mouse prostate cell lines showed that most of the prepared 1,2,3-triazolylpurine compounds were not cytotoxic.^40^ The 50% cytotoxic concentration of the tested derivatives was in the range from IC_50_ = 59.6 to 1528.7 μM, which indicated low cytotoxicity with negligible practical applicability. In turn, when aromatic amides of selected triterpenoids were studied in our team, agents with remarkable cytotoxicity were prepared, as demonstrated by several papers from the most recent period.^33,41−43^ Therefore, such molecules require a more detailed investigation, regarding designing their structures and exploring their pharmacological characteristics.

## 7 Purine Derivatives Constructed for Nitric Oxide Release

**Figure 13:**
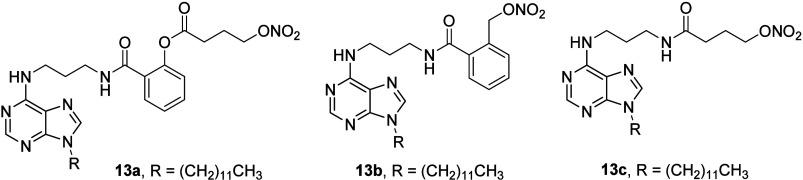
Structures of compounds **13a**–**13c**.

Nitric oxide (NO) displays effects as a cytotoxic agent against tumors, but its gaseous nature and short half-life hinder direct administration to tumor tissues.^44,45^ Therefore, it was necessary to design convenient compounds, in which NO may be accommodated as a part of their molecules.^46^ The purine scaffold offered such an opportunity, and novel 6,9-disubstituted purine derivatives, designed to ensure sustained NO release, were synthesized and investigated.^46^ Their significant antiproliferative, antimigratory, and anticlonogenic effects in HepG2 cell lines were studied, highlighting the NO release as a potent effector for treating hepatocellular carcinoma. The original study represented an effort to deliver exogenous NO *in situ* as an effective anticancer therapeutic.^46^ However, it seems that the literature data regarding the therapeutic potential of NO have mostly been controversial, and the authors decided that novel and extensive studies are required to further establish the physiological effect of NO.^46^ From the three prepared and studied compounds (**13a**–**13c**; Figure 13), **13a** was found to be a potent inhibitor of cancer cell proliferation, metastasis, and colonial growth due to the optimum release of NO.^46^ However, the mechanism of the process can be diverse owing to the reactivity of NO with inorganic molecules (transition metals and oxygen), prosthetic groups, or DNA structures. Such a reactivity could be ascribed to either cGMP-dependent or cGMP-independent physiological pathways.^47^ Several studies have reported different mechanisms, like inhibition of HIF-1a,^48^ mitochondrial respiration,^49^ and or DNA synthesis for anticancer effects.^46−48^ The investigation resulted in the conclusion that **13a** offered a long-lasting, controlled release of NO by first-order kinetics.^46^ The compound **13a** showed cytotoxicity in HepG2 cells (IC_50_ = 31.20 ± 5.75 μM) and NIH3T3 cells (IC_50_ = 70.49 ± 6.24 μM), and it inhibited the migration of cancer cells and colony formation. These important findings achieved with **13a** represented the immense potential to further fine-tune the *in vivo* NO release, mechanism of action, and translational efficacy as an anticancer therapeutic agent.^46^

## 8 Purine Derivatives for Targeting Proteolysis

**Figure 14:**
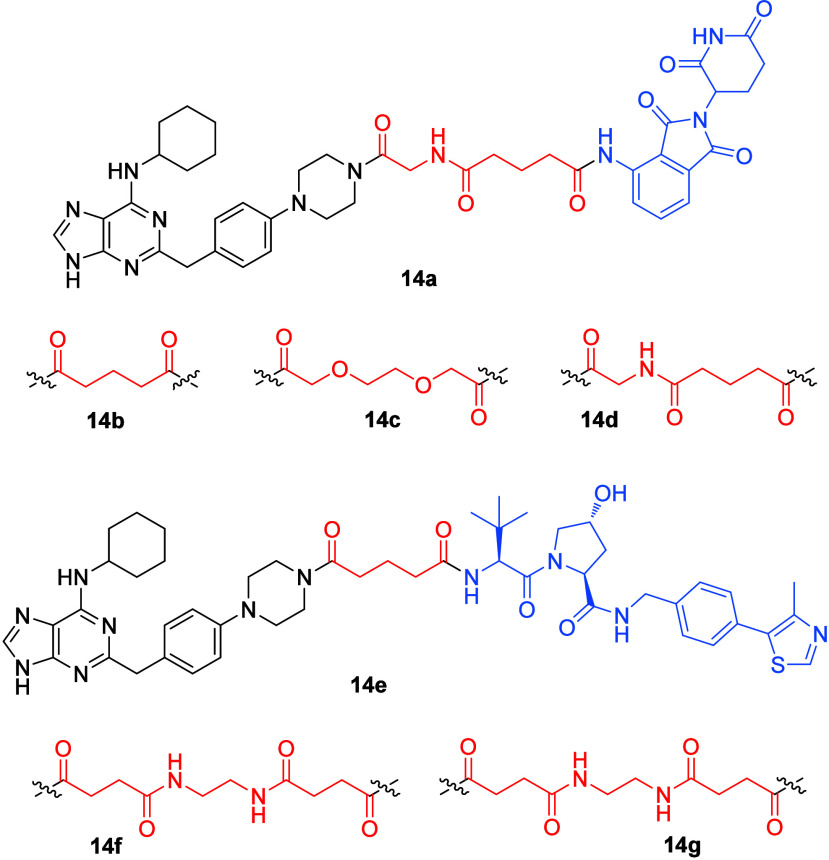
Structure of the most active compound **14a** accompanied by several other structures (**14b**–**14g**) that differ from **14a** by the structure of the linker drawn in the red color and either pomalidomide or the VHL ligand drawn in the blue color as the ligands for E3-ligase recruitment. The ligand for E3-ligase recruitment in **14d** is methyl pomalidomide, and the compound was used as a negative control.

The purine scaffold was employed recently in the development of 2-aminoadenine-based proteolysis-targeting chimeras as potent degraders of monopolar Spindle 1 kinase (TTK) and Aurora kinases (AURK) A and B. All these kinases are known as critical regulators of mitosis, and they play an important role in the progression of various types of cancer. The authors reported the design, synthesis, and biological evaluation of a series of compounds (chimeras) targeting the proteolysis of TTK and AURKs.^50^ Various degrader molecules were synthesized, based on four different 2-aminoadenine-based ligands, inducing either cereblon (CRBN) or von Hippel–Lindau (VHL) ligands for E3-ligase recruitment. The investigation resulted in the finding that the nature of the linker and the modification of the ligand significantly influenced the target specificity and degradation efficacy (**14a**–**14g**; Figure 14).^50^ Among the most potent degraders, **14a** (using pomalidomide as the ligand for E3-ligase recruitment) demonstrated a robust proteasome-mediated degradation of TTK with *D*_max_ = 66.5% and DC_50_ (6 h) = 17.7 nM, as compared to its structurally related inhibitor negative control **14d**, bearing the same linker as **14a** but using methyl pomalidomide as the ligand for E3-ligase recruitment (Figure 14).

The HiBiT data of the azareversine-based chimeras **14a**–**14g** after 6 h of treatment are summarized in Table 12. The effects of the synthesized compounds on AURKA, AURKB, and TTK mitotic kinases were assessed in cellular systems.^50^ The HiBiT cell lines were generated for all three mitotic kinases. A small, 11 amino acid-containing peptide fragment of luciferase (HiBiT) was fused to the coding sequence of the proteins and stably transduced in the MV4-11 cell line (MV4-11^AURKA-HiBiT^, MV4-11^AURKB-HiBiT^, and MV4-11^HiBiT-TTK^). Immunoblots showed a slightly higher expression of the protein in the corresponding HiBiT cell line compared with the parental MV4-11 cells. Even though separate bands were not observed for the HiBiT-tagged and endogenous untagged protein, a higher expression in HiBiT cell lines demonstrated the expression of HiBiT-tagged protein.^50^ In summary, the data demonstrate that **14a** showed the highest efficacy. The compounds **14a**–**14c**, bearing pomalidomide as the ligand for E3-ligase recruitment, displayed higher effects than **14e**–**14g**, bearing the VHL ligand as the ligand for E3-ligase recruitment.^50^

**Table 12:** HiBiT Data of Azareversine-Based Chimeras **14a**–**14g** after 6 h of Treatment^50^

	AURKB^a^	AURKA^b^	TTK^c^	AURKB^a^	AURKA^b^	TTK^c^
comp.	*D*_max_ [%]^d^	*D*_max_ [%]^d^	*D*_max_ [%]^d^	DC_50_ [nM]^e^	DC_50_ [nM]^e^	DC_50_ [nM]^e^
**azar.**^f^	44.53	52.60	47.70	79.33	99.50	118.00
**14a**^g^	43.60	78.80	66.50	570.25	108.67	17.67
**14b**^g^	30.60	68.40	27.70	380.00	94.00	3.00
**14c**^g^	36.10	68.90	39.40	598.00	239.00	66.00
**14d**^h^	48.30	55.25	32.55	363.00	299.00	600.00
**14e**^i^	33.80	55.60	32.70	2282.00	684.00	1963.00
**14f**^i^	inact.	28.30	28.40	-	9840.00	5651.00
**14g**^i^	29.10	39.50	34.20	2559.00	6503.00	1893.00

a MV4-11^AURKB-HiBiT^ cells.

b MV4-11^AURKA-HiBiT^ cells.

c MV4-11^HiBiT-TTK^ cells.

d *D*_max_: maximal degradation; compounds with degradation less than 15% are reported as “inactive”.

e DC_50_: half-maximal degradation concentration, calculated with the dose–response (four parameters) equation; compounds with degradation less than 25% were not calculated and reported as “-”.

f Azareversine.

g Ligand for the E3-ligase recruitment = pomalidomide.

h Ligand for the E3-ligase recruitment = methyl pomalidomide.

i Ligand for the E3-ligase recruitment = VHL-ligand.

## 9 Structure–Activity Relationship Analysis

The review of the most recent papers dealing with compounds based on the substituted purine scaffold clearly showed the importance of the applied substituents on the studied cytotoxicity. Compounds bearing the piperazine motif in the organic molecules mostly displayed high cytotoxicity in the studied cancer cell lines.^8−10,18^ However, a part of these structures showed high toxicity in the nonmalignant reference cells.^16^ This finding clearly indicates that additional substituents used in combination with the piperazine motif may enhance or reduce the final cytotoxicity of the studied compounds. The most successful compounds bearing the piperazine motif were the compounds **1j**, **1l**, **1m**, **1n**, and **1q** (Figure 1; Table 1) and the compounds **5e** (IC_50_ = 1.70 μM; AsPC-1 cell line), **5p** (IC_50_ = 4.56 μM; MIA-PaCa-2 cell line), **5q** (IC_50_ = 4.11 μM; MIA-PaCa-2 cell line), and **5r** (IC_50_ = 3.08 μM; MIA-PaCa-2 cell line) (Figure 5; Table 5). The compounds **1j**, **1l**, **1m**, **1n**, and **1q** complexed with Alk, BTK, and DDR2, and their binding site interactions and their binding affinities were analyzed by molecular docking and molecular dynamics simulations. The compounds **1j** and **1q** displayed similar interactions with the activation loop of the kinases. However, only compound **1j** reached the active sites of the kinases, and the cell cycle and signaling pathway analyses exhibited that only compound **1j** decreased phospho-SRC, phospho-Rb, cyclin E, and Cdk2 levels in liver cancer cells and induced apoptosis.

The compounds of the series **3a**–**3e** (Figure 3, Table 3) and **4a**–**4i** (Figure 4, Table 4) represent additional successful series of compounds derived from the purine scaffold.^10,16^ Compound **3d** was the most active one of the former series of compounds. Nevertheless, the whole series of compounds **3a**–**3e** (Figure 3, Table 3) was subjected to more detailed tests in a panel of several drug-sensitive hepatocellular carcinoma cell lines.^10^ The compounds **4a**–**4i** (Figure 4, Table 4) were tested for their ability to inhibit the recombinant Abl1 kinase, indicating **4b** as the most active compound of the latter series of compounds.^16^

The compounds of the series **2a**–**2g** (Figure 2, Table 2), **6a**–**6c** (Figure 6), and **7a**–**7j** (Figure 7, Table 6) showed only medium cytotoxicity values in different types of cancer cell lines, which made comparing them based on the structure–activity relationship analysis impossible.^9,21,27^

Metal coordination showed an enhancing effect on cytotoxicity in several compounds.^30,32^ Four complexes of fludarabine (**9c**–**9f**; Figure 9), bearing the *trans*-[Br(PPh_3_)_2_]Pt/Pd fragment, were investigated. The results revealed that the platinum complexes of fludarabine were more cytotoxic than their palladium analogues (Table 8). The platinum complexes of fludarabine showed IC_50_ < 10 μM in the cells of various solid tumor entities, including cisplatin-resistant ones, presumably due to the 10-fold higher cellular uptake of the platinum complexes. However, the palladium complexes of fludarabine interacted more readily with the isolated calf thymus DNA.^30^ Therefore, further investigation of both platinum and palladium complexes of fludarabine should be done in the future.

The alkaline earth metal (Mg^2+^, Ca^2+^, Sr^2+^, and Ba^2+^) complexes of guanine were also investigated for their potential anticancer and antibacterial effects.^32^ The *in vitro* cytotoxicity testing revealed that the alkaline earth metal complexes of guanine exhibited potential cytotoxic activity, showing LC_50_ = 18.55–40.61 μg·mL^–1^. A cervical cancer cell line (HeLa) was used to investigate the cytotoxic effects of the metal complexes of guanine, and each complex demonstrated cytotoxicity in the HeLa cell line.^32^ However, no guanine–metal complex displayed higher cytotoxicity than cisplatin.^32^ The antimicrobial and antifungal effects were also studied with these guanine complexes prepared with alkaline earth metals, resulting in the finding that guanine itself exhibited no antibacterial activity; however, its metal complexes showed significant antimicrobial and antifungal effects with no selectivity among the tested microorganisms.^32^

Hybrid molecules (conjugates) that combine characteristics of their components also seem to be convenient chemicals for future investigation, giving potential to discover novel structures with enhanced pharmacological potential by combining the characteristics of their components.^34,38,50^ The results of the studies performed with several types of hybrid molecules, often displaying supramolecular characteristics, confirm the therapeutic potential of such compounds and materials (Figures 10 and 14, Tables 9, 10, and 12).^34,38,50^ Providing any quantitative structure–activity relationship analysis is not possible because the authors of the different papers used different targets for their studies.^34,38,50^

However, the controlled release of NO from organic hybrid molecules, in which exogenous NO was bound, has not fully met the expectations up to now.^46^ Analogously, the presence of the purine scaffold in the cytokinin mimics resulted in a nonpreferred direction in the investigation of pharmacologically important purine derivatives.^21,38^ Therefore, a more detailed investigation of such structures will be required in a future.

Undoubtedly, an enhancing effect on cytotoxicity appeared when nanoassemblies were formed, using a biologically active compound (Figures 8 and 14, Tables 7 and 12).^28,50^ Nanovesicles and other nanoassemblies either may act as nanocarriers of a biologically active component that enable transporting of the biologically active agent to the target tissues or may be composed of a biologically active agent combined with convenient supporting species, mostly biopolymers, capable of forming the required nanoassemblies. In the latter case, the target nanoassemblies mostly show enhanced biological activity. In summary, nanoassemblies of different types, namely, those which show adequate biocompatibility, seem to become tools for more and more advanced technologies in preparing highly biologically active agents for targeted cancer treatments. A recently published review dealing with hybrid nanomaterials represents just a part of a broad field focusing on biological activity–nanoassembly relationship studies.^51^ The potential of aromatic nitrogen-bearing heterocycles that include purine derivatives capable of forming nanoassemblies in aqueous media should become a key direction in the investigation of these type of compounds, their conjugates, and potential hybrid nanomaterials self-composed from the relevant source molecules.

## 10 Conclusions and Future Challenges

A critical evaluation of the biological data in this review should be mentioned in this conclusion. The different authors cited herein used different cancer cell lines in their investigations and often applied different methods for testing their compounds. Due to that reason, there are factors that may affect comparability of the cytotoxicity values presented in this review in a negative way. Moreover, the results of the investigations published in this field were achieved by various research groups and in various geographical regions. All these factors make comparability of the results presented by different research groups, as well as the structure–activity relationship analysis, extremely difficult. The presented results from all over the world are valuable for representing the source values for a more detailed future investigation of purine scaffold-based pharmacologically active agents. However, to consider the practical application of purine-based agents for the potential treatment of different types of cancer, preferably a single team should focus on the most active compounds produced by different research groups to finally obtain comparable results. That idea may become a future challenge of researchers or pharmaceutical companies to bring the selected and outstanding results of basic research into clinical practice.
